# Cyclodextrin derivatives decrease Transient Receptor Potential vanilloid 1 and Ankyrin 1 ion channel activation via altering the surrounding membrane microenvironment by cholesterol depletion

**DOI:** 10.3389/fcell.2024.1334130

**Published:** 2024-02-28

**Authors:** Andrea Kinga Nehr-Majoros, János Erostyák, Éva Fenyvesi, Edina Szabó-Meleg, Levente Szőcs, György Sétáló, Zsuzsanna Helyes, Éva Szőke

**Affiliations:** ^1^ Department of Pharmacology and Pharmacotherapy, Medical School and Centre for Neuroscience, University of Pécs, Pécs, Hungary; ^2^ National Laboratory for Drug Research and Development, Budapest, Hungary; ^3^ Hungarian Research Network, Chronic Pain Research Group, Pécs, Hungary; ^4^ Department of Experimental Physics, Faculty of Sciences, University of Pécs, Pécs, Hungary; ^5^ János Szentágothai Research Centre, University of Pécs, Pécs, Hungary; ^6^ CycloLab Cyclodextrin Research and Development Ltd., Budapest, Hungary; ^7^ Depatment of Biophysics, Medical School, University of Pécs, Pécs, Hungary; ^8^ Department of Medical Biology, Medical School, University of Pécs, Pécs, Hungary; ^9^ PharmInVivo Ltd., Pécs, Hungary

**Keywords:** lipid raft, cyclodextrin, cholesterol, TRPA1, TRPV1, pain, neurogenic inflammation

## Abstract

Transient Receptor Potential Vanilloid 1 (TRPV1) and Ankyrin 1 (TRPA1) are nonselective cation channels expressed in primary sensory neurons and several other non-neuronal structures such as immune cells, keratinocytes, and vascular smooth muscle cells. They play important roles in nociception, pain processing and their chanellopathies are associated with the development of several pathological conditions. They are located in cholesterol- and sphingolipid-rich membrane lipid raft regions serving as platforms to modulate their activations. We demonstrated earlier that disruption of these lipid rafts leads to decreased TRP channel activation and exerts analgesic effects. Cyclodextrins are macrocyclic molecules able to form host-guest complexes with cholesterol and deplete it from the membrane lipid rafts. The aim of this study was to investigate 8 structurally different (methylated and non-methylated) CD derivatives on cell viability, mitochondrial membrane potential, membrane composition and activation abilities of the TRPV1 and TRPA1 channels. We showed that non-methylated derivatives have preferable safety profiles compared to methylated ones. Furthermore, methylated derivatives reduced mitochondrial membrane potential. However, all investigated derivatives influence the ordered cell membrane structure depleting membrane cholesterol and inhibit the TRPV1 agonist capsaicin- and the TRPA1 agonist allyl isothiocyanate-induced Ca^2+−^influx. This mechanism of action might provide novel perspectives for the development of peripherally acting analgesics via indirectly decreasing the generation and transmission of nociceptive signals.

## 1 Introduction

Transient Receptor Potential Vanilloid 1 (TRPV1) and Ankyrin 1 (TRPA1) are thermosensitive members of the TRP superfamily. These non-selective cation channels are expressed on primary sensory neurons and peripheral nerve terminals, where their activation is triggered by mechanical, chemical, or thermal stimuli (Moran et al., 2011; Vay et al., 2012). TRPV1 is gated by noxious heat (T > 43°C), acidic conditions (pH < 6.0), lipophilic compounds (endogenous arachidonic acid or other fatty acid metabolites) or vanilloid-type (capsaicin: CAPS, resiniferatoxin: RTX) and other (anandamide, oxytocin, etc.) chemical substances (Hwang et al., 2000; Smart et al., 2000; Helyes et al., 2003; Raisinghani et al., 2005; Bianchi et al., 2006a), while TRPA1 is activated by noxious cold (T < 17°C), and numerous endogenous or exogenous compounds (allyl isothiocyanate (AITC, mustard oil), menthol, formaldehyde, etc.) (Story et al., 2003; Jordt et al., 2004; Bianchi et al., 2006a; Trevisani et al., 2007; Szolcsányi and Sándor, 2012; Szolcsányi and Pintér, 2013; Moore et al., 2018; Benítez-Angeles et al., 2020). These ion channels show high co-expression, and both are sensitized by various inflammatory mediators (bradykinin, prostaglandins, histamine, etc.) (Salas et al., 2009; Akopian, 2011; Grace et al., 2012; Wilzopolski et al., 2021). Both TRPV1 and TRPA1 were identified as potential targets in pain management (Helyes et al., 2003; Bölcskei et al., 2005; Moran and Szallasi, 2017; Demartini et al., 2018; Souza Monteiro de Araujo et al., 2020; Yeh et al., 2020; Iftinca et al., 2021; Tajti et al., 2023). They play crucial roles in pain sensation and transmission, furthermore, local release of pro-inflammatory neuropeptides (calcitonin gene-related peptide (CGRP), Substance P) from peripheral nerve endings upon their activation induces vasodilation and plasma protein extravasation (Szolcsányi, 2004; Szallasi et al., 2007; Luo et al., 2013; Laing and Dhaka, 2016). This so-called neurogenic inflammation is proven to be involved in numerous inflammatory pathological conditions accompanied with chronic pain (arthritis, psoriasis) (Sousa-Valente and Brain, 2018; Maglie et al., 2021). Most of the recent therapies do not reduce the neurogenic component of pain (Helyes et al., 2003; Szolcsányi and Sándor, 2012; Kaneko and Szallasi, 2014; Nilius and Szallasi, 2014; Seidel et al., 2022; Wang and Thyagarajan, 2022). Thus, further discoveries are required to reveal new potential molecular mechanisms for analgesia with reliable effect. TRPV1 and TRPA1 are intensively investigated members of the superfamily, but their direct antagonism in human studies was challenging due to potential severe side effects (Kaneko and Szallasi, 2014; Moran and Szallasi, 2017). Therefore, finding novel perspectives for the modulation of TRPV1 and TRPA1 activation is still necessary.

TRPV1 and TRPA1 are localized in special microdomains of the cell membrane (Liu et al., 2006; Szolcsányi, 2008). These so-called lipid rafts are nanoscale (10–200 nm) dynamic structures particularly rich in cholesterol, sphingolipids, gangliosides, and other special membrane components due to lateral segregation of these constituents in the cell membrane based on liquid-liquid immiscibility (Simons and Ikonen, 1997; Simons and Sampaio, 2011; Sonnino and Prinetti, 2013). This composition increases membrane stiffness and makes raft regions more ordered compared to other membrane areas, serving as a platform to receptor complexation and facilitating receptor activation (Mishra and Joshi, 2007; Bobkov and Semenova, 2022). Therefore, disruption of the lipid rafts, decreasing their integrity and targeting hydrophobic lipid-protein interactions seem to be an efficient novel mechanism of action for decreasing the level of activation of raft-associated receptors (Szoke et al., 2010; Sághy et al., 2015; Horváth et al., 2020; Horváth et al., 2021; Sántha et al., 2020).

Cyclodextrins (CDs) are non-reducing cyclic oligosaccharides composed of α-D-glucopyranose units. In their most common native forms, 6, 7 or 8 glucopyranose units are linked together by α-1,4 glycosidic bonds to form a ring shape, called alpha-, beta- or gamma-CD, respectively (Szejtli, 1998). Their unique truncated cone structure, with hydrophilic outer surface and hydrophobic inner cavity allows the formation of host-guest inclusion complexes with hydrophobic molecules, thus increasing water solubility of the guest molecules. This host-guest complex formation is dependent on the size of the guest molecule and the cavity size of the CDs, which is considered to have an opening of 4.5–5.3 Å, 6.0–7.0 Å and 7.5–8.5 Å for α-, β- and γ-CDs, respectively and similar cavity depth for all forms (∼7.9 Å) (Das et al., 2020). Benefiting from this property CDs are frequently used in the pharmaceutical industry as carriers for solubilizing compounds and to increase stability or bioavailability (Loftsson and Brewster, 2011; Puskás et al., 2023). By attaching functional groups to different members of the glucopyranose ring, a variety of derivatives with different chemical structures and biological activities can be obtained from the native forms. Some derivatives are already used themselves as active pharmaceutical ingredients (Takazawa et al., 2016; Ishitsuka et al., 2022), but active research is being conducted on the use of CDs in newer indications (Kovacs et al., 2022).

We previously demonstrated that methyl-beta-cyclodextrin (MCD), the gold standard in lipid raft research is effective in depleting membrane cholesterol *in vitro* in primary cultures of trigeminal (TG) neurons and in Chinese hamster ovary (CHO) cell line, and we also described that MCD treatment decreased TRPV1 and TRPA1 receptor activation via lipid raft disruption. We have provided evidence that MCD induced cholesterol depletion and changed the membrane polarity and fluidity in TG neurons and CHO cells (Szoke et al., 2010; Sághy et al., 2015). We proved that MCD pretreatment had analgesic effect via TRPV1 in and TRPA1 receptor inhibition, as it significantly reduced the number of CAPS-activated eye-wiping movements and formalin-evoked acute nocifensive behavior in mouse models, however it did not influence RTX-induced thermal or mechanical allodynia (Horváth et al., 2020).

Considering the relatively high cytotoxicity of MCD our aim in this study was to investigate the effects of 8 CD derivatives with different structures on cell viability, mitochondrial membrane potential related to their safety, as well as membrane cholesterol and lipid composition, and TRPV1 and TRPA1 receptor activation.

## 2 Materials and methods

### 2.1 CD derivatives and other chemicals

CD derivatives offered by CycloLab Cyclodextrin Research and Development Ltd. were the following: (2-Hydroxypropyl)-gamma-cyclodextrin (HPGCD), (2-Hydroxypropyl)-beta-cyclodextrin (HPBCD), Sulfobutylether-beta-cyclodextrin sodium salt (SBECD), (2-Hydroxy-3-N,N,N-trimethylamino)propyl-beta-cyclodextrin (QABCD), Randomly methylated beta-cyclodextrin (RAMEB), Heptakis(2,6-di-O-methyl)-beta-cyclodextrin isomeric mixture of 50% and 95% isomeric purity (DIMEB-50 and DIMEB-95, respectively), Heptakis(2,3,6-tri-O-methyl)-beta-cyclodextrin (TRIMEB). The molecular formula and characteristics of the CD derivatives are summarized in Table 1. CD solutions were always freshly prepared prior to treatment dissolving appropriate amount of CD derivatives in the solvent used in the respective experiment (sterile complete Dulbecco’s Modified Eagle’s Medium (DMEM), extracellular solution (ECS) or Ca^2+^-free Hank’s solution). Laurdan, DMSO and Filipin III were purchased from Sigma-Aldrich, Inc. (St. Louis, MO, United States). Laurdan was dissolved in DMSO to reach a 10 mM stock solution, further dilutions were performed in ECS. Supplementary Table S1 contains the detailed list and catalogue/product numbers of the applied reagents.

**TABLE 1:** Main characteristics of the investigated CD derivatives.

Cyclodextrin derivative	Abbreviation	Molecular formula	Chemical structure, degree of substitution (DS, n)	Characteristics
(2-Hydroxypropyl)-gamma-cyclodextrin	HPGCD	C_48_H_80-n_O_40_ · (C_3_H_7_O)_n_	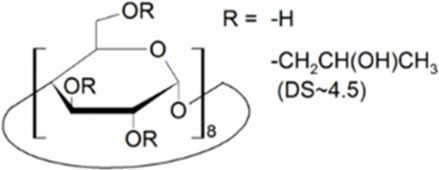	Non-methylated, neutral
(2-Hydroxypropyl)-beta-cyclodextrin	HPBCD	C_42_H_70-n_O_35_ · (C_3_H_7_O)_n_	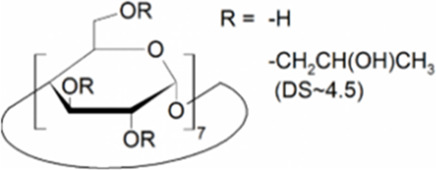	Non-methylated, neutral
Sulfobutylether-beta-cyclodextrin sodium salt	SBECD	C_42_H_70-n_O_35_ · (C_4_H_8_O_3_SNa)_n_	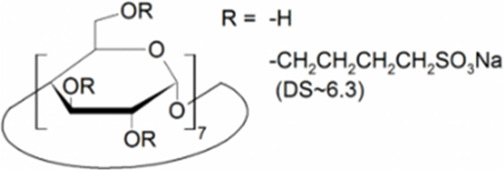	Non-methylated, anionic
(2-Hydroxy-3-N,N,N-trimethylamino)propyl-beta-cyclodextrin	QABCD	C_42_H_70-n_O_35_ · (C_6_H_15_ONCl)_n_	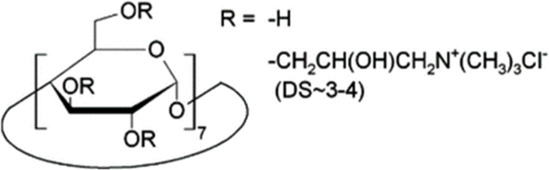	Non-methylated, cationic
Randomly methylated beta-cyclodextrin	RAMEB	C_42_H_70-n_O_35_ · (CH_3_)_n_	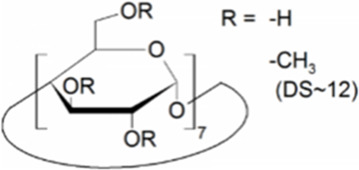	Methylated, neutral
Heptakis(2,6-di-O-methyl)-beta-cyclodextrin of 50% isomeric purity	DIMEB-50	C_42_H_70-n_O_35_ · (CH_3_)_n_	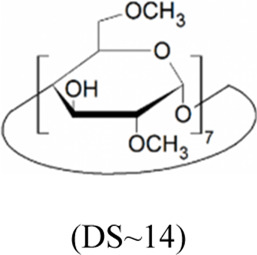	Mehtylated, neutral, isomeric purity: ∼50%
Heptakis(2,6-di-O-methyl)-beta-cyclodextrin of 95% isomeric purity	DIMEB-95	C_42_H_70-n_O_35_ · (CH_3_)_n_	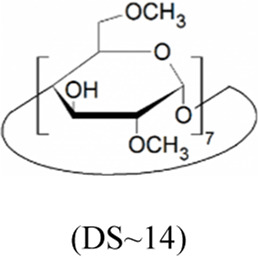	Mehtylated, neutral, isomeric purity: ∼95%
Heptakis(2,3,6-tri-O-methyl)-beta-cyclodextrin	TRIMEB	C_63_H_112_O_35_	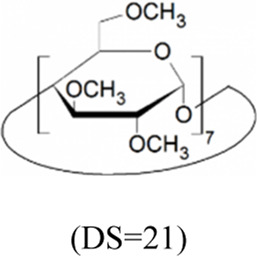	Methylated, neutral

### 2.2 Cells and culturing

Native and TRPA1 or TRPV1 receptor-expressing CHO cells were cultured in complete DMEM (500 mL low glucose (1 g/L) DMEM (Capricorn Scientific GmbH, Germany) supplemented with 50 mL fetal bovine serum (Euroclone, Italy), 5 mL GlutaMAX™-I (100X) solution (Thermo Fisher Scientific, Waltham, MA, United States), 5 mL Gibco™ MEM non-essential amino acid solution (100X) and 500 µL penicillin-streptomycin mixture (Lonza Group, Switzerland)) solution. Cells were grown in T-25 size cell culture flasks under standard cell culture conditions (37°C, 5% CO_2_) and passaged at 70%–80% confluency. To avoid *Mycoplasma* infection, regular monitoring was performed by qPCR technique. The detailed description of the *Mycoplasma* screening method is added as Supplementary Material.

### 2.3 Viability assay

The effect of CD treatment in different concentrations on native CHO cells’ viability was investigated using CellTiter-Glo^®^ Luminescent Cell Viability Assay (Promega Corporation, WI, United States) according to the manufacturer’s protocol. This method is based on measuring the intracellular ATP level of live cells. Briefly, native CHO cells were seeded to opaque 96-well culture plates at a density of 5,000 cells/100 µL complete DMEM per well. Side rows and columns of the plate were not used but filled with phosphate buffer saline (PBS) to avoid artefacts. Cells were grown overnight in a cell culture incubator (37°C, 5% CO_2_). The next day, the culture medium was aspirated from the cells and replaced with increasing concentrations of CD solutions in sterile complete DMEM (100 µL/well). Untreated cells served as control, and complete DMEM control was used to detect a luminescent background. Plates were incubated for 24 h in a cell culture incubator, then equilibrated at room temperature for 30 min before the end of the incubation time. At the end of the incubation period, 100 µL of room temperature CellTiter-Glo reagent was added to each well, then plates were placed on an orbital shaker for 2 min and incubated for an additional 10 min at room temperature without shaking. The luminescent signal was measured using an EnSpire AlphaLisa microplate reader (Perkin Elmer, Inc., Waltham, MA, United States). Luminescent values were normalized and the results from the treated cells were compared to that of the untreated control.

### 2.4 Mitochondrial membrane potential analysis

MitoTracker™ Red CMXRos fluorescent staining (Thermo Fisher Scientific, Waltham, MA, United States) was used to investigate the effect of the CD derivatives on the mitochondrial membrane potential of native CHO cells. 5,000 cells/100 µL complete DMEM per well were seeded onto 10-well CELLview™ cell culture slides and incubated under standard conditions (37°C, 5% CO_2_) overnight. The next day cells were treated for 24 h (37°C, 5% CO_2_) with CD solutions dissolved in sterile complete DMEM. After washing with PBS, cells were stained with 50 µL of 250 nM (37 °C) MitoTracker™ Red CMXRos stain (30 min, 37°C, 5% CO_2_), washed with PBS and 100 µL complete DMEM without phenol red was added to the cells followed by immediate examination. Z-stacks were taken in cells’ total depth applying 1 µm stepsize using a Zeiss LSM 710 laser scanning confocal microscope (Carl Zeiss Microscopy, Jena, Germany) equipped with Plan-Apochromat 63×/1.4oil objective. For excitation 594 nm laser line was used, emission wavelength was 645 nm. Data quantification was performed using ImageJ software (National Institutes of Health, United States) mean fluorescence intensity values of CD-treated cells were compared to untreated control cells (minimum 80–120 cells from minimum 3 independent experiments).

### 2.5 Investigation of cholesterol depletion

The cholesterol-binding macrolide antibiotic Filipin III originated from the bacterial strain *Streptomyces filipinensis* is a commonly used fluorescent dye for visualizing cholesterol intracellularly (Králová et al., 2018; Bruno et al., 2023). Native CHO cells were grown on 10-well CELLview™ cell culture slides at a density of 5,000 cells/100 µL complete DMEM per well. CD derivatives were dissolved in extracellular solution (ECS) in appropriate concentrations. The next day, after PBS washing, media was aspired from the cells, cells were washed with PBS and incubated in 100 µL of ECS (control) or 100 µL of CD solution dissolved in ECS for 1 h (37°C, 5% CO_2_). Cells were fixed with 4% paraformaldehyde (PFA, 100 µL/well) for 1 h, and incubated with 100 µL of glycine in PBS (1.5 mg/mL) at room temperature. Cholesterol was stained with 100 µL of Filipin III solution (0.05 mg Filipin III/mL PBS/10% FBS) for 2 h at room temperature. Cells were washed three times with PBS after every step. Finally, cells were covered with ProLong™ Glass Antifade Mountant (Invitrogen, Waltham, MA, United States). Staining was visualized using an Olympus Fluoview-1000 system. Micrographs were generated using an Olympus Fluoview-1000 system on an Olympus IX81 microscope stage equipped with an Olympus DP70 digital camera and through an Olympus UPlan FL N, Phase2 objective (40×/0.75), with mercury lamp DAPI filters (excitation: 335–375 nm, emission: 440–500 nm). Image size was set at 680 × 512 and ISO at 200. Mean pixel intensity per cell was measured in ImageJ software. Background correction was performed by subtracting the mean intensity of the non-cell-containing background of the same sized area from the mean intensity value of each cell. Mean intensity values of CD-treated cells were compared to untreated control cells (minimum 150 cells from minimum 3 independent experiments).

### 2.6 Membrane order and polarization detection

The detection of fluorescence emission from the Laurdan dye (6-dodecanoyl-2-N,N-dimethyl-2-naphthylamine) is an effective method for studying the membrane structure of living cells as well as model membranes. The polarity-sensitive fluorescent dye Laurdan can detect the shift from liquid ordered to liquid disordered phase of the membrane microenvironment. The excitation and emission spectra of a fluorescence probe embedded in the membrane phospholipid bilayer provide information on the fluidity as well as the orderliness of the membrane environment surrounding the Laurdan molecule (Golfetto et al., 2013). For sample preparation, native CHO cells were seeded in a 12-well cell culture plate at a density of 150,000 cells/1 mL complete DMEM per well. After overnight incubation (37°C, 5% CO_2_) the supernatant of the treated samples was replaced with 1 mL of CD solution dissolved in sterile complete DMEM in the appropriate concentration in duplicates and incubated for 45 min (37°C, 5% CO_2_). After washing with PBS, 1 mL of 80 µM Laurdan solution in ECS was added to the cells for 40 min (37°C, 5% CO_2_), and after three washing steps with PBS, cells were carefully harvested with a cell scraper in 1–1 mL of PBS. Perkin Elmer LS50B Luminescence Spectrometer (Perkin Elmer Inc., Waltham, MA, United States) equipped with a pulsed Xenon lamp was applied to record fluorescence emission spectra and polarization spectra. All measurements were carried out in a 4 mm path length quartz cuvette (Hellma 104F-QS). Temperature was kept at 20 °C during the fluorescence measurements. To quantify the spectral changes, Generalized Polarization function (GP) is widely used. From emission data we get the emission GP function: ${GP}_{\text{emi}}=\frac{I_{435}\;-\;I_{500}}{I_{435}+I_{500}}$ , where *I*_435_ and *I*_500_ stand for the intensities in fluorescence emission spectrum at 435 nm and 500 nm, respectively. Please note, that “Generalized Polarization” has no connection to any optical polarization. This quantity is used in describing spectral changes and the definition is analogous to the definition of the physical quantity “polarization”. Higher value of *GP* means more ordered, and lower value of *GP* means more disordered state of membrane. Anisotropy is defined as $r=\frac{I_{\text{VV}}‐G*I_{\text{VH}}}{I_{\text{VV}}+2*G*I_{\text{VH}}}$ , where *G* is the spectrofluorometer’s sensitivity factor given by $G=\frac{I_{\text{HV}}}{I_{\text{HH}}}$ . Subscripts H and V describe horizontal or vertical polarization of excitation (first letter) or emission (second letter) lights, respectively.

### 2.7 Radioactive ^45^Ca^2+^uptake to detect TRPV1 and TRPA1 receptor activation

Radioactive ^45^Ca^2+^-uptake measurement is used to investigate the effect of CD derivatives on receptor activation and can detect the combined response of thousands of cells in count per minute (CPM). CHO cells stably expressing TRPA1 or TRPV1 receptors were plated at a density of 4,000 cells/well in 15–15 μL complete DMEM in 72-well mini culture dishes and incubated overnight under standard cell culture conditions (37°C, 5% CO_2_). The next day, adhered cells were washed five times with Ca^2+^-free Hank’s solution, followed by treatment with increasing concentrations of CD solutions (37°C, 30 min). Following another five times of washing with Ca^2+^-free Hank’s solution the cells were incubated in a mixture of 10 μL of test solutions (200 µM TRPA1 agonist allyl isothiocyanate (AITC) or 100 nM TRPV1 agonist capsaicin (CAPS)) and 200 μCi/mL ^45^Ca^2+^ isotope dissolved in 10 μL Ca^2+^-free Hank’s solution for 2 min at room temperature. After washing five times with ECS solution, the mini culture dish containing the cells was dried at 75 °C and lysed with 15 μL of 0.1% sodium dodecyl sulfate (SDS) to collect the accumulated ^45^Ca^2+^ isotope. Radioactivity of the lysate was measured in 2 mL scintillation liquid using a Packard Tri-Carb 2800 TR counter as CPM. The values of treated samples were compared to those measured in untreated control cells.

### 2.8 Statistical analysis

In all cases, results were obtained from a minimum of three independent experiments. Data were evaluated using GraphPad Prism 8.0.1 (GraphPad, La Jolla, CA, United States) or OriginPro 8.5 (OriginLab Corporation, Northampton, MA, United States) software. Statistical analysis was performed using D’Agostino-Pearson normality testing, followed by one-way ANOVA or Kruskal–Wallis test to compare control and treated groups, with Tukey’s or Dunnett’s *post hoc* test. Microscopic images were evaluated using ImageJ 1.53 software.

## 3 Results

### 3.1 Methylated, but not non-methylated CD derivatives reduce CHO cell viability

**FIGURE 1:**
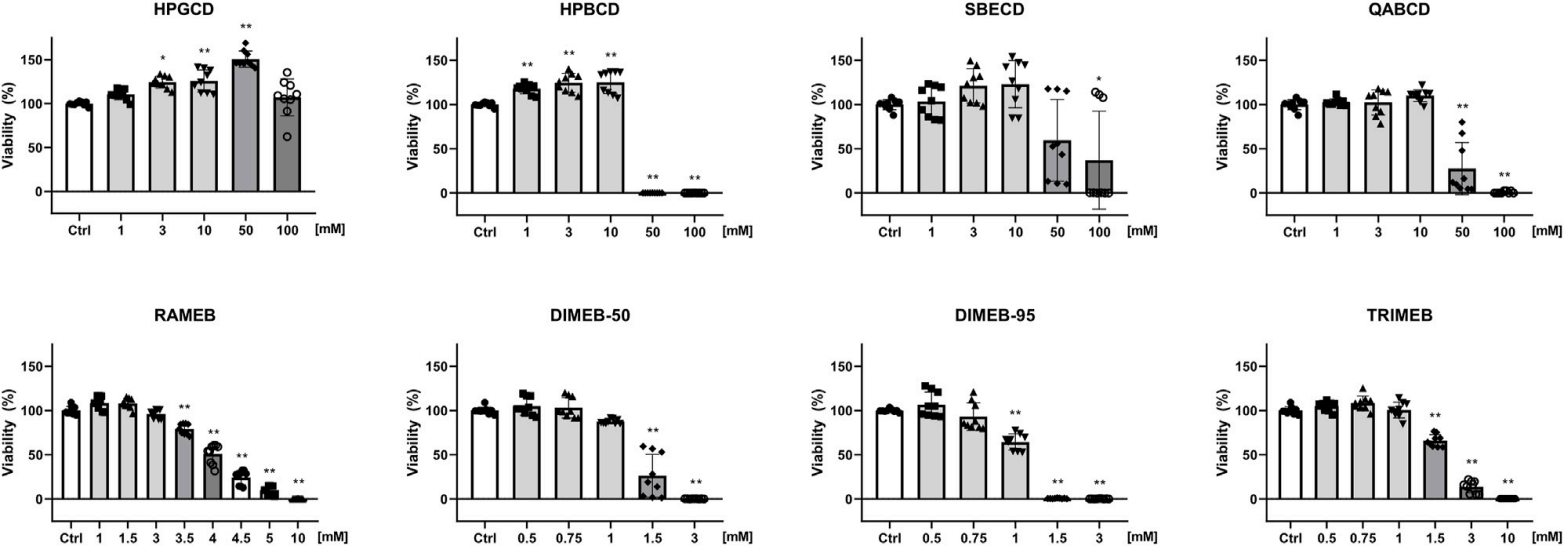
Effect of CD derivatives on the viability of native CHO cells. The percentage values of viable cells after 24-h CD treatment are presented as means ± SD of three independent experiments in biological triplicates (n = 9, one-way ANOVA, Tukey *post hoc* test; *p < 0.001, **p < 0.0001 vs control).

Non-methylated CD derivatives (HPGCD, HPBCD, QABCD, SBECD) did not influence CHO cell viability up to 10 mM concentration CD in the CellTiter-Glo^®^ Luminescent Cell Viability Assay, in which the luminescent signal was proportional to the amount of ATP produced by living cells. The gamma derivative HPGCD was found to be safe even at higher concentrations (50, 100 mM), while methylated derivatives were cytotoxic at lower concentrations, in the order DIMEB-95 (0.75 mM) > DIMEB-50 (1 mM) = TRIMEB (1 mM) > RAMEB (3 mM) (Figure 1). Significantly elevated luminescent signal was obtained for hydoxypropylated derivatives HPGCD and HPBCD.

### 3.2 Certain non-methylated CD derivatives increase, methylated derivatives decrease the mitochondrial membrane potential of CHO cells

**FIGURE 2:**
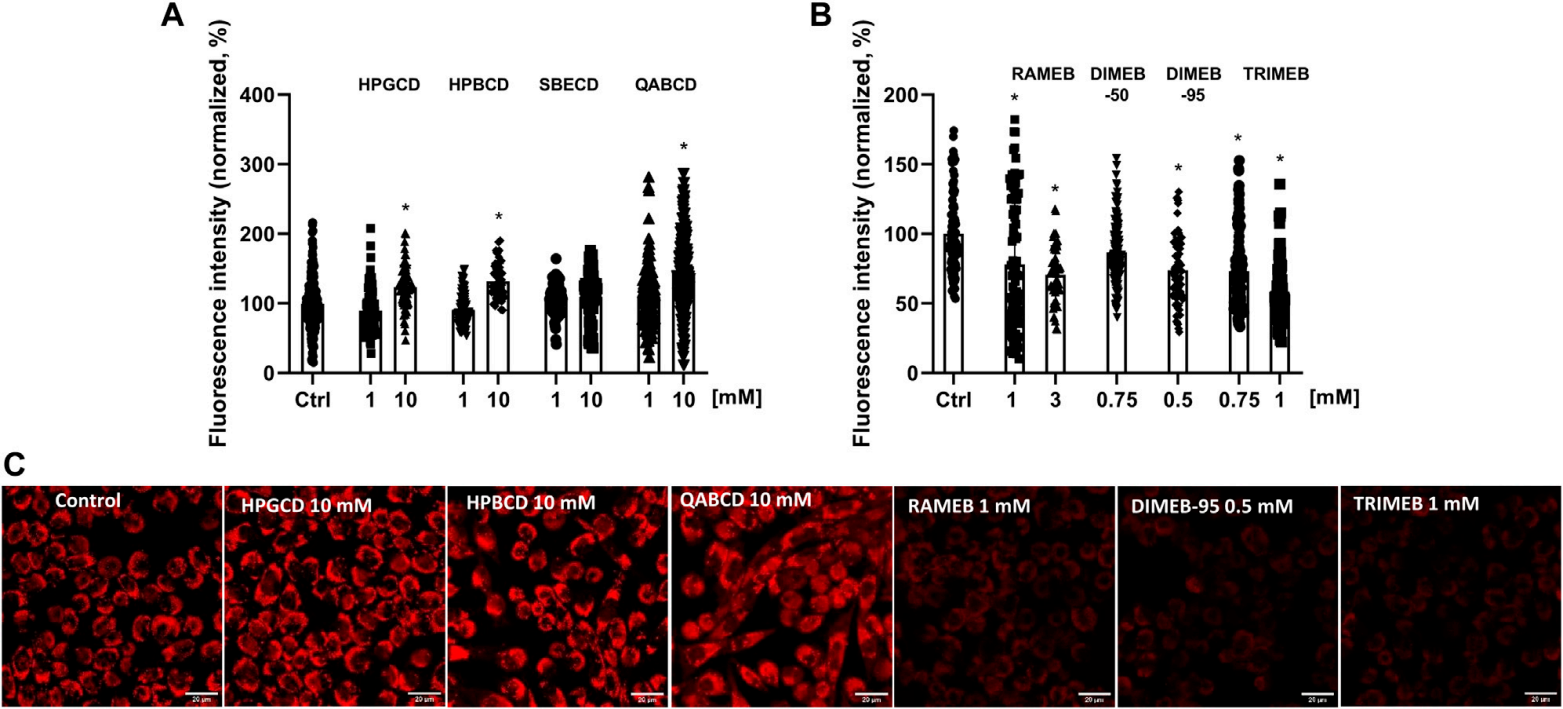
Alterations of mitochondrial membrane potential of native CHO cells after 24-h CD treatment. The percentage values of fluorescence intensity changes after 24-h treatment with non-methylated **(A)** and methylated **(B)** CDs are shown as means ± SD of 80–120 cells from min. 3 independent experiments (Kruskal–Wallis test, Dunn’s multiple comparison test; *p < 0.0001 vs control). Representative images showing the mitochondria of control cells compared to 24-h CD-treated ones in the case of statistically significant samples, scale bar: 20 µm **(C)**.

In mitochondrial membrane potential measurements CD derivatives were tested at concentrations not affecting cell viability. The intensity of the MitoTracker Red™ CMXRos fluorescent dye staining actively functioning mitochondria significantly increased to 123%, 132% and 143% after 24-h HPGCD, HPBCD and QABCD treatment (10 mM), respectively. Meanwhile, methylated derivatives decreased fluorescence intensity even at concentrations not influencing viability, except for DIMEB-50 (Figure 2). Following 3 mM RAMEB, 0.75 mM DIMEB-95 and 1 mM TRIMEB treatment the intensity of the MitoTracker Red™ CMXRos dye decreased by 29%, 27% and 43% compared to control cells, respectively.

### 3.3 All CD derivatives reduce cholesterol content of CHO cells

**FIGURE 3:**
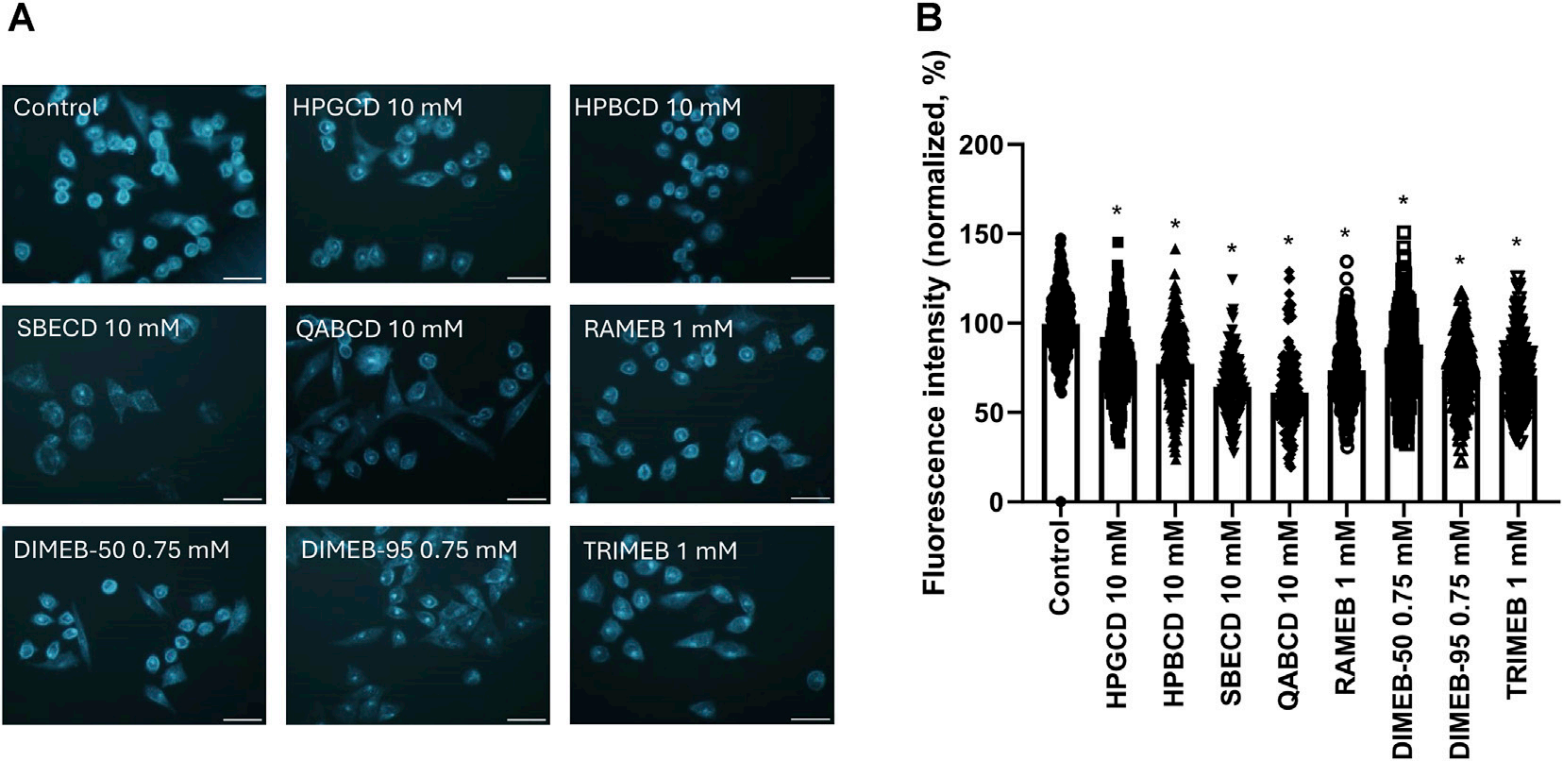
Effect of 1-h CD treatment on the cholesterol content of native CHO cells. Representative images after Filipin III staining of untreated (control) and CD-treated cells **(A)**. Quantification of fluorescent signals of the Filipin III dye **(B)**. Normalized fluorescence intensity values are presented in percentage as means ± SD of 150–200 CHO cells from min. 3 independent experiments (Kruskal–Wallis test, Dunn’s multiple comparison test; *p < 0.0001 vs control). Scale bar: 40 µm.

To investigate the cholesterol-depleting ability of CD derivatives, filipin staining of untreated control CHO cells was compared to that of CHO cells treated with CD solutions at concentrations not affecting cell viability. Filipin stain showed accumulation in both the membrane and perinuclear compartments of control cells, indicating the presence of cholesterol. All CD derivatives significantly reduced the signal of filipin staining demonstrating cholesterol depletion (Figure 3). 10 mM HPGCD, 10 mM HPBCD, 10 mM SBECD, 10 mM QABCD, 3 mM RAMEB, 0.75 mM DIMEB-50, 0.75 mM DIMEB-95 and 1 mM TRIMEB treatment resulted in a decrease of the cholesterol content of cells to 79%, 77%, 64%, 61%, 73%, 79%, 73% and 70% *versus* untreated control cells (100%), respectively.

### 3.4 CD derivatives disintegrate membrane structure, with DIMEB-50 being the most efficient

**FIGURE 4:**
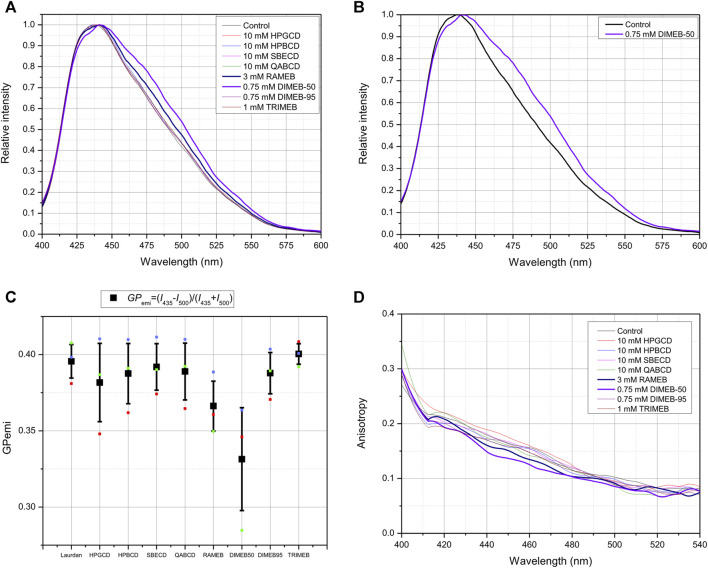
Effect of 45-min CD-treatment on the membrane lipid order and polarization of native CHO cells. Normalized Laurdan emission spectra (λ_exc_ = 370 nm) of native CHO cells labeled with 80 µM Laurdan. CD-treated cells are compared to untreated control cells **(A)**. Normalized Laurdan emission spectra (λ_exc_ = 370 nm) of DIMEB-50 compared to native CHO cells labeled with 80 µM Laurdan **(B)**. Quantification of spectral changes as GP function calculated from emission data of three independent experiments, presented as means ± SD. *GP*_emi_=(*I*_435_−*I*_500_)/(*I*_435_ + *I*_500_), where *I*_435_ and *I*_500_ stand for the intensities in fluorescence emission spectrum at 435 and 500 nm, respectively **(C)**. Anisotropy *r* is defined as *r=*(*I*_vv_-G*I_VH_)/(*I*_VV_+2*G*I_VH_), where *G* is the spectrofluorometer’s sensitivity factor given by: *G* = *I*_HV_/*I*_HH_, where *I*_HV_ and *I*_HH_ are measured using horizontally polarized excitation and vertically and horizontally polarized emission, respectively **(D)**.

In disordered phase, the incorporated Laurdan molecules in the membrane are more likely to be surrounded by water molecules, which quenches the fluorescence, while a red shift is observed in the shape of fluorescent emission spectra (lower *GP* value). Following CD treatment by DIMEB-50 and RAMEB gave the most remarkable spectral red-shifts (Figures 4A,B) indicating higher dielectric constant and more disordered membrane microenvironment, confirmed by their outlying *GP* values (Figure 4C). It is worth mentioning that a slight shift towards L_d_ state is also discernable in cases of all other CD derivatives compared to control.

The same tendency can be seen on fluorescence anisotropy spectra: lower anisotropy values in cases of DIMEB-50- and RAMEB-treated samples mean less restricted rotational movement of Laurdan, that is related to the loss off membrane stiffness compared to control cells (Figure 4D).

### 3.5 CD derivatives decrease the activation of TRPV1 and TRPA1 receptors *in vitro*

**FIGURE 5:**
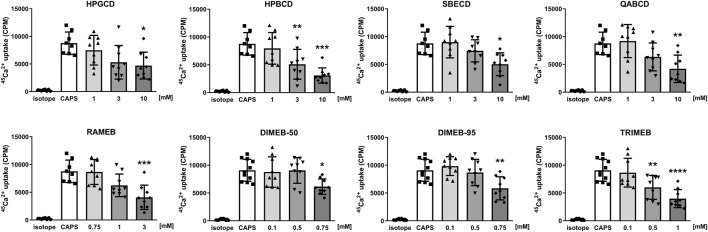
Effect of CD derivatives on radioactive ^45^Ca^2+^-uptake of TRPV1-expressing CHO cells following activation with 200 nM CAPS presented as counts per minute (CPM). Isotope-induced ^45^Ca^2+^-uptake was used as control (isotope, 200 μCi/mL). Each column represents the mean ± SD of three independent experiments in biological triplicates (n = 9, one-way ANOVA, Dunnett’s *post hoc* test, *p < 0.05; **p < 0.01; ***p < 0.001 vs isotope control).

**FIGURE 6:**
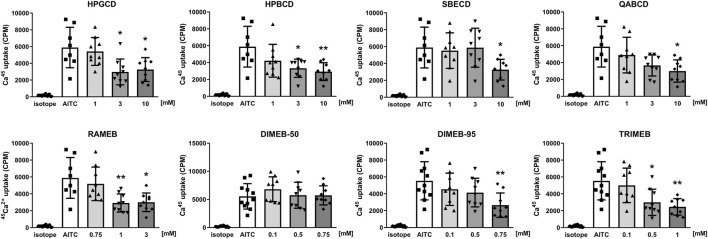
Effect of CD derivatives on radioactive ^45^Ca^2+^-uptake of TRPA1-expressing CHO cells following activation with 200 µM AITC presented as counts per minute (CPM). Isotope-induced ^45^Ca^2+^-uptake was used as control (isotope, 200 μCi/mL). Each column represents the mean ± SD of three independent experiments in biological triplicates (n = 9, one-way ANOVA, Dunnett’s *post hoc* test, *p < 0.05; **p < 0.01; ***p < 0.001 vs isotope control).

TRPV1 and TRPA1 receptor activations were evaluated by measuring the overall radioactive ^45^Ca^2+^ uptake response of cells in response to 100 nM CAPS and 200 µM AITC, respectively. CD treatments significantly reduced TRPV1 receptor activation in a concentration-dependent manner in cases of all tested CD derivatives (Figure 5). Similarly, CD derivatives at the highest applied concentration inhibited ^45^Ca^2+^ uptake in response to TRPA1 receptor activation, except for DIMEB-50 (Figure 6). 10 mM HPGCD, HPBCD, SBECD, QABCD, 3 mM RAMEB, 0.75 mM DIMEB-50 and DIMEB-95, as well as 1 mM TRIMEB diminished CAPS-evoked TRPV1 activation to 53%, 33%, 56%, 42%, 45%, 67%, 61% and 42% and AITC-induced TRPA1 activation was reduced to 52%, 46%, 53%, 48%, 49%, 100%, 44% and 48% *versus* control cells (100%), respectively.

## 4 Discussion

These are the first results showing that different CD derivatives inhibit TRPV1 and TRPA1 ion channel activation *in vitro*, presumably via lipid raft disruption resulting from membrane cholesterol depletion and influencing the ordered cell membrane structure.

We determined the concentration of each CD derivative, which does not yet influence cell viability. This effect is structure-dependent: non-methylated beta derivatives (HPBCD, SBECD, QABCD) are less cytotoxic being safe up to 10 mM concentration, the gamma derivative HPGCD is not toxic even at higher concentration (100 mM). The 0.1–10 mM concentration range of CDs was selected based on the literature (Kovacs et al., 2022). Carradori et al. used HPBCD in 0.5–10 mM concentration range for detecting its effect on intracellular cholesterol accumulation in NPC-like fibroblasts, while 1,333 mg/kg and 4,000 mg/kg doses were applied to achieve the therapeutic effect in NPC1 mice (Carradori et al., 2020). In cellular Niemann Pick type C models 0.1 mM–1 mM HPBCD and MCD effectively reduced the cholesterol levels (Rosenbaum et al., 2010). In another study Kilpatrick et al. found that 1 mM HPBCD can control lysosomal functions in Parkinson model cells (Kilpatrick et al., 2015). On the other hand, Kiss et al. found that HPBCD did not hemolyse the human blood cells up to 200 mM concentration (Kiss et al., 2010). Our viability studies clearly showed the biological relevance of the millimolar CD concentrations. These data are in accordance with previous results stating that the cytotoxicity of CD derivatives depends on the cavity size, and this is in connection with the higher cholesterol depleting capacity of beta derivatives compared to gamma derivatives. The characteristics of the substituted functional group are also crucial, as hydroxypropylation or addition of an ionic group to the CD ring results in derivatives with more favorable safety profiles. Methylated derivatives decreased cell viability at lower concentrations, in the order DIMEB-95 (0.75 mM) > DIMEB-50 (1 mM) = TRIMEB (1 mM) > RAMEB (3 mM), which data support earlier findings observed on other cell lines: the presence of methyl groups increases cytotoxicity, the extent of which is also influenced by the number and the position of the methyl groups (Kiss et al., 2007; Kiss et al., 2010; Róka et al., 2015; Szente et al., 2018; Rassu et al., 2020). Interestingly, in the case of HPGCD and HPBCD at certain concentrations we observed significantly elevated luminescent signal, which was proportional to the amount of ATP produced by living cells. It has been reported that MCD treatment increased mitochondrial ATP production in vascular endothelial cells (Yamamoto et al., 2020). MCD has been described to induce G1 phase cell cycle arrest in macrophages (Choi et al., 2004), which may lead to reduced absolute cell number without a significant cell death rate, and since the arrested cells continue to grow, mitochondrial content and ATP synthesis per cell also increase (Chan et al., 2013).

When investigating the alterations of mitochondrial membrane potential of CHO cells in response to CD treatment at non-toxic concentrations, we demonstrated that 10 mM HPGCD, HPBCD and SBECD increased mitochondrial membrane potential. RAMEB (1 and 3 mM), DIMEB-95 (0.5 mM) and TRIMEB (1 mM) treatment resulted in decreased fluorescent signal, indicating reduced mitochondrial membrane potential and potential mitochondrial dysfunction in the treated cells. According to previous findings, the methylated derivative MCD impaired the function of rat liver mitochondria, but it was able to partially inhibit CaCl_2_-induced mitochondrial swelling (Ziolkowski et al., 2010). Furthermore, the investigation of the effect of different CD derivatives on human keratinocytes revealed cytochrome *c* release following native BCD and MCD treatment, supporting the idea that the function of the mitochondrial electron transport chain and ATP synthesis were affected. In the same experimental setup HPBCD treatment led to only a slight release of cytochrome *c* and was found to be relatively non-noxious (Schönfelder et al., 2006).

Concerning structural effects of CD treatments on the cell membrane, we previously showed by the cholesterol-binding Filipin staining that MCD and a novel carboxamido-steroid compound depleted cholesterol from the plasma membrane of CHO cells (Szoke et al., 2010; Sághy et al., 2018). In the present experiments we demonstrated that all investigated CD derivatives significantly removed membrane cholesterol, reaching reduced cholesterol content of 61%–79% compared to the control, with the ionic derivatives SBECD and QABCD being slightly more potent in the applied concentration, than the methylated derivatives RAMEB, DIMEB-95 and TRIMEB, followed by hydroxypropylated derivatives, where HPGCD and HPBCD had almost the same cholesterol depletion ability. It is important to highlight, that this is a subjective comparison, as concentrations of methylated and non-methylated derivatives are highly different in the experiments, as they were chosen according to viability results to ensure that the applied treatment does not influence cell viability. Comparing the two DIMEBs, DIMEB-50 showed lower cholesterol depletion than the purer isomer DIMEB-95, propounding that 2,6-DIMEB has more significance in cholesterol depletion than other DIMEB isomers (2,3-, and 3,6-DIMEB and some over- and undermethylated isomers). DIMEB-95 has a similar effect as RAMEB and TRIMEB, which derivatives were applied in slightly higher concentration, suggesting that 2,6-DIMEB is also more potent cholesterol extractor in this cellular system than TRIMEB containing only one isomer 2,3,6-TRIMEB or RAMEB consisting of more components, but not containing 2,6-DIMEB as shown by their chromatograms (Fenyvesi et al., 2014a). The exact mechanism and stoichiometry of CD-cholesterol depletion, as well as complexation in case of different derivatives have been actively studied (Williams et al., 1998; López et al., 2011; Kucáková and Dolensky, 2020). Although based on the cavity size beta derivatives forming 2:1 CD:cholesterol complexes are considered to be the most suitable guests for cholesterol, in cellular environment gamma derivatives forming 1:1 CD:cholesterol complexes also showed similar cholesterol depleting capacity (Szente et al., 2018; Yamada et al., 2021). The picture becomes even more complex if we also take into consideration the heterogeneity of the cell membrane and the possible complexation of CDs with other cellular and membrane components potentially influencing this system (Huang and London, 2013; Litz et al., 2016; Hammoud et al., 2019; Abe and Kobayashi, 2021; Geda et al., 2022). Additionally, the ability of immortalized human epithelial Caco-2 cells to take up CDs from solutions by mechanisms differing from simple diffusion was also described (Fenyvesi et al., 2014b).

Steady-state fluorescence emission and anisotropy spectra measurements showed plausible evidence of a more disordered membrane structure in cases of DIMEB-50 and RAMEB treated samples. In the case of other derivatives there was also a shift towards the liquid disordered phase in the cell membrane, but the difference was less pronounced. Both the spectral changes of fluorescence emission spectra (lower *GP* values) and anisotropy spectra (lower *r* values) indicate more disordered membrane structures. These data coincide with previously published results in similar experiments concerning the loss of lipid raft integrity induced by treating cells with other lipid raft disruptors like MCD, the carboxamido steroid C1, myriocin and higher concentration of resolvins (Sághy et al., 2015; Sághy et al., 2018; Payrits et al., 2020; Horváth et al., 2022). We demonstrated in our earlier study that cholesterol depletion by MCD treatment increased membrane fluidity in CHO cells. In the present study, the difference between results after DIMEB-50 and DIMEB-95 treatments, which contain 2,6-DIMEB as main component (with 50% and 95% isomeric purity, respectively) suggests the importance of the contribution of other components that are present in DIMEB-50 in a higher ratio. These results also raise the question of CD derivatives’ complexation and interaction with other building blocks of the raft regions.

We are the first to show the inhibitory effect of the investigated CD derivatives on TRPV1 and TRPA1 ion channel activation. CD derivatives concentration-dependently decreased CAPS- and AITC-induced TRPV1 and TRPA1 activation and consequent ^45^Ca^2+^-uptake. DIMEB-50 was the only derivative which did not induce significant inhibition on TRPA1 ion channel. In general, it can be concluded that methylated derivatives have higher potency on TRPV1 and TRPA1 channel activation. These results support our previous findings that cholesterol depletion by MCD or the carboxamido-steroid C1 resulted in significantly decreased TRP ion channel activation both *in vitro* and *in vivo* (Szoke et al., 2010; Sághy et al., 2018; Horváth et al., 2020). The crucial role of membrane cholesterol content in TRP ion channel activation and the potential importance of direct cholesterol-TRP interactions were also described by others (Startek et al., 2019; Startek and Talavera, 2020). The importance of the lipid microenvironment surrounding the TRP channels in regulating their conformation between active, inactive or desensitized states has been recently visually demonstrated by cryo-electron microscopy (Zhang et al., 2021). These results support our hypothesis that inhibition of TRP ion channels may either be due to a change in their position in the plasma membrane making some binding sites unattainable for activators or some other direct protein-lipid hydrophobic interactions. Our research group also described, that on CHO cells the effect of lipid raft disruption was different in cases of different vanilloid type agonists having distinct binding sites on the TRPV1 ion channel, e.g., the effect of CAPS but not resiniferatoxin was diminished by MCD (Szoke et al., 2010). The regulatory function of the lipid nano-environment on various transmembrane proteins, receptors and ion channels has been also extensively studied elsewhere, emphasizing the fact that several direct and indirect factors must be considered as well (Zakany et al., 2020; Levental and Lyman, 2023). In addition, the impact of direct receptor-CD interactions on receptor function has also been proposed for some ion channels, e.g., TWIK-related acid-sensitive potassium channel 1 (TASK-1), TASK-3 and voltage gated potassium channels (K_v_) (Zakany et al., 2020; Kovacs et al., 2022). This should also be examined in further investigations in the case of TRPA1 and TRPV1 channels as well.

In conclusion, our findings confirm that alteration of the surrounding lipid microenvironment by depleting cholesterol from the cell membrane influences TRP channel function, and CDs are suitable molecules for this purpose. Since differences between the chemical structure of different CD derivatives have consequences on their biological effects, the appropriate CD derivative must be chosen carefully. We found that the number and position of methyl groups on a CD molecule may influence the cholesterol depleting effect and cytotoxicity. Even the less cytotoxic non-methylated CD derivatives diminish TRP ion channel activation via modification of the plasma membrane integrity. Even if we consider the potential systemic toxicity of high dose CD treatment, they might have drug developmental potentials mainly as peripheral analgesics in pathological conditions such as chronic pain involving these ion channels.

## Data Availability

The raw data supporting the conclusion of this article will be made available by the authors, without undue reservation.
