# Supplementary material for: Cyclodextrin derivatives decrease Transient Receptor Potential vanilloid 1 and Ankyrin 1 ion channel activation via altering the surrounding membrane microenvironment by cholesterol depletion
Source: Front Cell Dev Biol. 2024 Feb 28;12:1334130. doi: 10.3389/fcell.2024.1334130 (PMC10933000; doi:10.3389/fcell.2024.1334130)
Supplement: Supplementary file 1 [file Table1.DOCX]

| **Reagent** | **Manufacturer** | **Catalogue no./Product no.** |
| --- | --- | --- |
| Allyl isothiocyanate | Sigma-Aldrich, Inc. | 377430 |
| Capsaicin | Sigma-Aldrich, Inc. | M2028-1G |
| CellTiter-Glo® Luminescent Cell Viability Assay | Promega Corporation | G7572 |
| DIMEB-50 | CycloLab Cyclodextrin Research and Development Ltd. | CY-2004.0 |
| DIMEB-95 | CycloLab Cyclodextrin Research and Development Ltd. | CY-2004.3 |
| DMEM | Capricorn Scientific GmbH | DMEM-LPA |
| DMEM without phenol red | Sigma-Aldrich, Inc. | D4947-500ML |
| DMSO | Sigma-Aldrich, Inc. | D8418 |
| Fetal bovine serum | Euroclone (Italy) | ECS0196L |
| Filipin III | Sigma-Aldrich, Inc. | F4767 |
| GlutaMAX^TM^-I (100X) solution | Thermo Fisher Scientific | 35050-061 |
| Gibco^TM^ MEM non-essential amino acid solution (100X) | Thermo Fisher Scientific | 11140050 |
| HPBCD | CycloLab Cyclodextrin Research and Development Ltd. | CY-2005.2 |
| HPGCD | CycloLab Cyclodextrin Research and Development Ltd. | CY-3005.2 |
| Laurdan | Thermo Fisher Scientific | D250 |
| MitoTracker™ Red CMXRos | Thermo Fisher Scientific | M46752 |
| Penicillin-streptomycin mixture | Lonza Group | 17-602E |
| ProLong™ Glass Antifade Mountant | Invitrogen | P36980 |
| QABCD | CycloLab Cyclodextrin Research and Development Ltd. | CY-2009 |
| RAMEB | CycloLab Cyclodextrin Research and Development Ltd. | CY-2004.1 |
| SBECD | CycloLab Cyclodextrin Research and Development Ltd. | CY-2041.2 |
| Sodium dodecyl sulfate | Sigma-Aldrich, Inc. | L3771 |
| TRIMEB | CycloLab Cyclodextrin Research and Development Ltd. | CY-2003 |

*Supplementary table 1.* The list of reagents applied in the experiments.

Supplementary methods:

Mycoplasma screening:
300 µL supernatant from cells was centrifuged (13000 rpm, 5 min) and DNA was extracted from the cells by adding 40 µL DNA-release and Dilution Buffer mixture. After 5 minutes incubation on room temperature the suspension was centrifuged (13000 rmp, 1 min), incubated on 98 °C, then centrifuged again (13000 rpm, 1 min). 1 µL of the supernatant containing the extracted DNA was mixed with 10 µL Phire Tissue PCR mastermix, 8 µL DNA- RNA-free water, 1 µL primer mix (10 µM GPO3 forward primer and 10 µM MGSO reverse primer). PCR reaction was performed, and products were separated by gel electrophoresis. DNA extracted from Mycoplasma infected CHO cells served as positive control and DNA- RNA-free water as negative control.

Viability measurement with trypan blue dye exclusion method:

Native CHO cells were seeded to 12-well cell culture plates at a density of 200,000 cells/1 mL complete DMEM per well. Cells were grown overnight in a cell culture incubator (37°C, 5% CO_2_). The next day, the culture medium was aspirated from the cells and replaced with indicated concentrations of CD solutions in sterile complete DMEM (100 µL/well). Untreated cells served as control. Plates were incubated for 24 hours in a cell culture incubator. Cells were trypsinized, then centrifuged (1000 rpm, 5 min) and 1 mL complete DMEM was added to the cells. Trypan blue dye was added to the cell suspension in 1:1 ratio. Viability was measured using Luna-II™ Automated Cell Counter (Logos Biosystems, Inc., VA, USA). The results from CD-treated samples were normalized to untreated control cells.

Supplementary results:

The effect of CD derivatives on the viability of CHO cells was investigated by trypan blue dye exclusion method to confirm the results of CellTiter-Glo Lumunescent Cell Viability Assay. CDs were applied in the concentration, which did not decrease cell viability in the ATP-based assay. 24-hour CD treatment resulted in 92.35-100 % viability results, meaning that cells could tolerate the 24-hour CD treatment.

| **CD derivative** | **Viability (%), normalized** |
| --- | --- |
| Control | 100.00 |
| HPGCD 10 mM | 99.37 |
| HPBCD 10 mM | 100.00 |
| SBECD 10 mM | 98.01 |
| QABCD 10 mM | 94.14 |
| RAMEB 3 mM | 97.78 |
| DIMEB-50 0.75 mM | 92.35 |
| DIMEB-95 0.75 mM | 97.08 |
| TRIMEB 1 mM | 96.58 |

*Supplementary table 2.* Normalized viability data of CHO cells following 24-hour CD treatment measured by trypan blue dye exclusion method. The experiment was performed in biological triplicates, table shows the average of 3 measurements.

| **Sample** | **Fluorescence intensity (a.u.)** | **SD** |
| --- | --- | --- |
| Control | 22,5 | 1,8 |
| HPGCD | 19,4 | 4,3 |
| HPBCD | 19,4 | 4,4 |
| SBECD | 18,1 | 3,0 |
| QABCD | 18,4 | 4,7 |
| RAMEB | 17,5 | 3,4 |
| DIMEB50 | 13,7 | 1,5 |
| DIMEB95 | 23,9 | 7,0 |
| TRIMEB | 22,0 | 2,1 |

*Supplementary table 3.* Effect of 45-min CD-treatment on the membrane lipid order and polarization of native CHO cells. Maximal fluorescence intensity values of native CHO cells labeled with 80 µM Laurdan averaged from 3 independent experiments. CD-treated cells are compared to untreated control cells.
